# Mitochondrial genome analysis of *Mylochromis lateristriga*

**DOI:** 10.1080/23802359.2015.1137814

**Published:** 2016-02-10

**Authors:** Xiao-Long Yuan, Zhong-Feng Zhang, Xin-Min Liu, Yong-Mei Du, Peng Zhang, Xiao-Dong Hou

**Affiliations:** Tobacco Research Institute of Chinese Academy of Agricultural Sciences, Qingdao, China

**Keywords:** Mitochondrial genome, *Mylochromis lateristriga*, phylogeny analysis.

## Abstract

*Mylochromis lateristriga* is a species of cichlid endemic and prefers sheltered bays or vegetated substrates. In this study, the mitochondrial genome of *M. lateristriga* was completed using PCR and Sanger sequencing method. The genome is a circular molecule with the length of 16 576 bp and contains 13 protein-coding genes (PCGs), 22 transfer tRNA genes and two ribosomal RNA genes. Except *COX1* gene, the other protein-coding genes are started with ATG. The average A + T content is 54.1%. Gene overlaps have been found at four junctions and have involved a total of 26 bp. Compared with other cichlid species, the genome exhibits high similarity in both the genome size and the genome structure. ML phylogenetic analysis was implemented with other 20 related species to verity the genetic relationship of *M. lateristriga*.

Mitochondria are founded in all eukaryotic cells (animals, photosynthetic organism, etc.), which originally comes from a single symbiotic event (Gary 2011). The mitochondrial DNA of vertebrates is a small, self-replicating and double-stranded circular molecule ranging in size from approximately 15 to 20 kb (Shadel & Clayton 1997). Recently, mitochondrial genomes made contribution to resolve relationships among cichlid species (Hulsey et al. 2013). Mylochromis *lateristriga,* belongs to cichlid, is a species endemic and prefers sheltered bays or vegetated substrates (Daget et al. 1984). However, there is no essential DNA molecular research for *M. lateristriga*, which become an obstacle when we want to investigate the evolutionary relationships with other closed species on complete mitochondrial DNA level. Hence, the mitochondrial genome of *M. lateristriga* was completed in order to investigate its genome structure, content and phylogenetic relationship with other freshwater species in this present study.

One individual of *M. Lateristriga* was purchased from Qingdao Nanshan market, PR China on 16 July 2014 and maintained in aerated seawater at 25°C for 1 week before processing. The adductor muscles were dissected and preserved in 95% ethanol and stored at −20°C. The mitochondrial genome of *M. Lateristriga* was completed using PCR and Sanger sequencing method. The genome is 16 576 bp in length. The whole genome was annotated at MitoFish website (http://mitofish.aori.u-tokyo.ac.jp/) (Iwasaki et al. 2013). Its genome comprises a circular DNA strand and encodes 13 protein genes (PCGs) which involved in the oxidative phosphorylation machinery (ND1, ND 2, ND 3, ND 4, ND 4L, ND 5 and ND 6), three subunits of the cytochrome c oxidase complex (COX1, COX2 and COX3), two subunits of ATP synthase (Atp 6 and Atp 8) and the cytochrome b subunit (Cyt b). ATG is the start codon of 12 protein-coding genes, while COX1 initiates with GTG. Also, it contains 22 transfer RNA genes and two ribosomal RNA genes (12S and 16S r RNA). The protein-coding genes, tRNA and r RNA regions occupied 66.78%, 9.2% and 15.86% of the whole genome, respectively. Additionally, gene overlaps have been found at four junctions and have involved a total of 26 bp. For example, the tRNA-Ile and ND1 shared five nucleotides in common, ND4L and ND4 share seven nucleotides, Atp 6 and Atp 8 overlap by 10 nucleotides and ND5 and ND6 by four nucleotides. The gene content and the structure are highly similar with other species of Lake Malawi cichlids (Hulsey et al. 2013). Mitogenome sequence of *M. lateristriga* has been deposited in Genbank under accession number KU056478.

In order to elucidate the evolution position of *M. lateristriga* in the phylogeny of fish, a global analysis was performed using 13 common protein sequences (ND1, ND2, ND3, ND4, ND4L, ND5, ND6, COX1, COX2 COX3, Atp6, Atp8 and Cytb) among *M. lateristriga* and other 20 related fish mitochondrial genomes (Figure 1). These genes were concatenated in a head-to-tail format using MAFFT with default settings (Katoh et al. 2005) and then these sequences were aligned using CLUSTAL X version 1.81 with the default settings (Thompson et al. 1997). Finally, the phylogenetic tree was constructed using RAxML version 8.1.12 (Stamatakis 2014) with GTR + I+G model. For each node, the bootstrap support was calculated using 1000 replicates. The species from Lake Malawi cichlids located in the same clade, consistent with their assignment. The ML tree presented *M. lateristriga* and *Diplotaxodon limnothrissa* formed a sister group and clustered in the family

**Figure 1.**
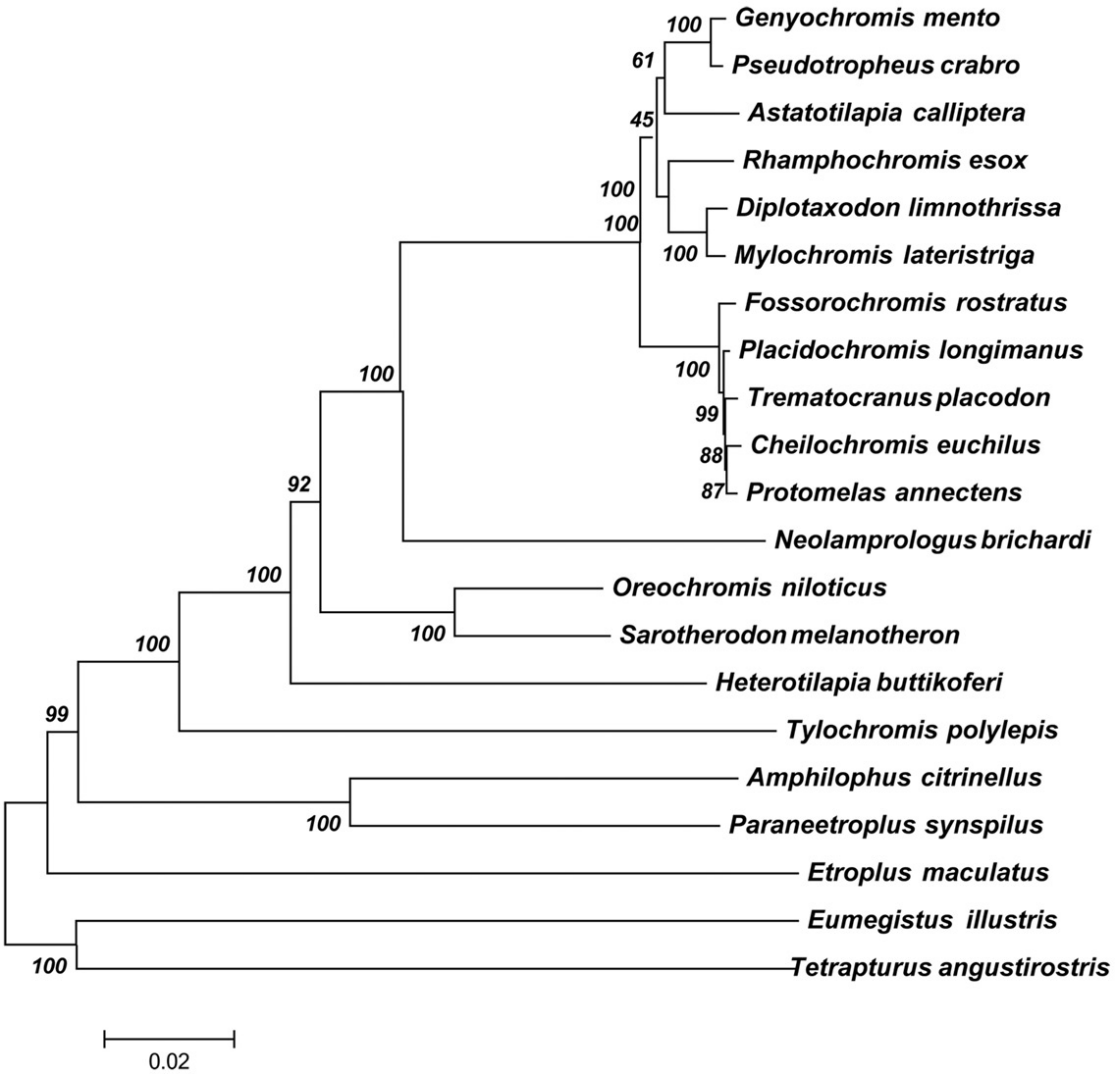
The phylogenetic relationships among 21 mitochondrial genomes. For each node, the bootstrap support was calculated using 1000 replicates. GenBank accession numbers of mitochondrial genomes used in this phylogeny analysis were listed: *Genyochromis mento *(JN628858); *Pseudotropheus crabro* (JN628854); *Astatotilapia calliptera* (JN628855); *Rhamphochromis esox* (JN628860); *Placidochromis longimanus *(KT309044); *Diplotaxodon limnothrissa *(JN628851); *Mylochromis lateristriga* (JN628851); *Fossorochromis rostratus* (KT290557); *Placidochromis longimanus* (KT309044); *Trematocranus placodon* (JN628850); *Cheilochromis euchilus* (JN252050); *Protomelas annectens* (KT188786); *Neolamprologus brichardi* (AP006014); *Oreochromis niloticus* (GU238433); *Sarotherodon melanotheron* (JF894132); *Heterotilapia buttikoferi* (KF866133); *Tylochromis polylepis* (AP009509); *Amphilophus citrinellus* (KJ081546); *Paraneetroplus synspilus* (KF879808); *Etroplus maculatus* (AP009505); *Eumegistus illustris* (AP012497); Tetrapturus angustirostris (AB470303).

Here we report the complete mitochondrial genome of *M. lateristriga*, and its total length and gene content were similar to those of other cichlid species, which not only can supply its mitochondrial information but also can make contribution to further understanding the evolutionary relationships of cichlid on mtgenome level.
